# Supplementary material for: SCALE: modeling allele-specific gene expression by single-cell RNA sequencing
Source: Genome Biol. 2017 Apr 26;18:74. doi: 10.1186/s13059-017-1200-8 (PMC5407026; doi:10.1186/s13059-017-1200-8)
Supplement: Supplementary file 1 — Figures S1–S15. Table S5. Supplementary Methods. (PDF 3472 kb) [file 13059_2017_1200_MOESM1_ESM.pdf]

Supplemental Data

Modeling allele-specific gene expression by single-cell RNA sequencing

Yuchao Jiang[1], Nancy R Zhang[2,*], Mingyao Li[3,*]

[1] Genomics and Computational Biology Graduate Program, Perelman School of Medicine, University of Pennsylvania, Philadelphia, PA 19104, USA

[2] Department of Statistics, The Wharton School, University of Pennsylvania, Philadelphia, PA 19104, USA

[3] Department of Biostatistics and Epidemiology, Perelman School of Medicine, University of Pennsylvania, Philadelphia, PA 19104, USA

* To whom correspondence should be addressed. Tel: (+1) 215-746-3916; Fax: (+1) 215-573-4865; Email: nzh@wharton.upenn.edu, mingyao@mail.med.upenn.edu

Figure S1. scRNA-seq protocol and technical variability. Dropouts and amplification and sequencing bias are introduced in library preparation and sequencing. These technical variability needs to be adjusted for accurate and unbiased downstream analysis.

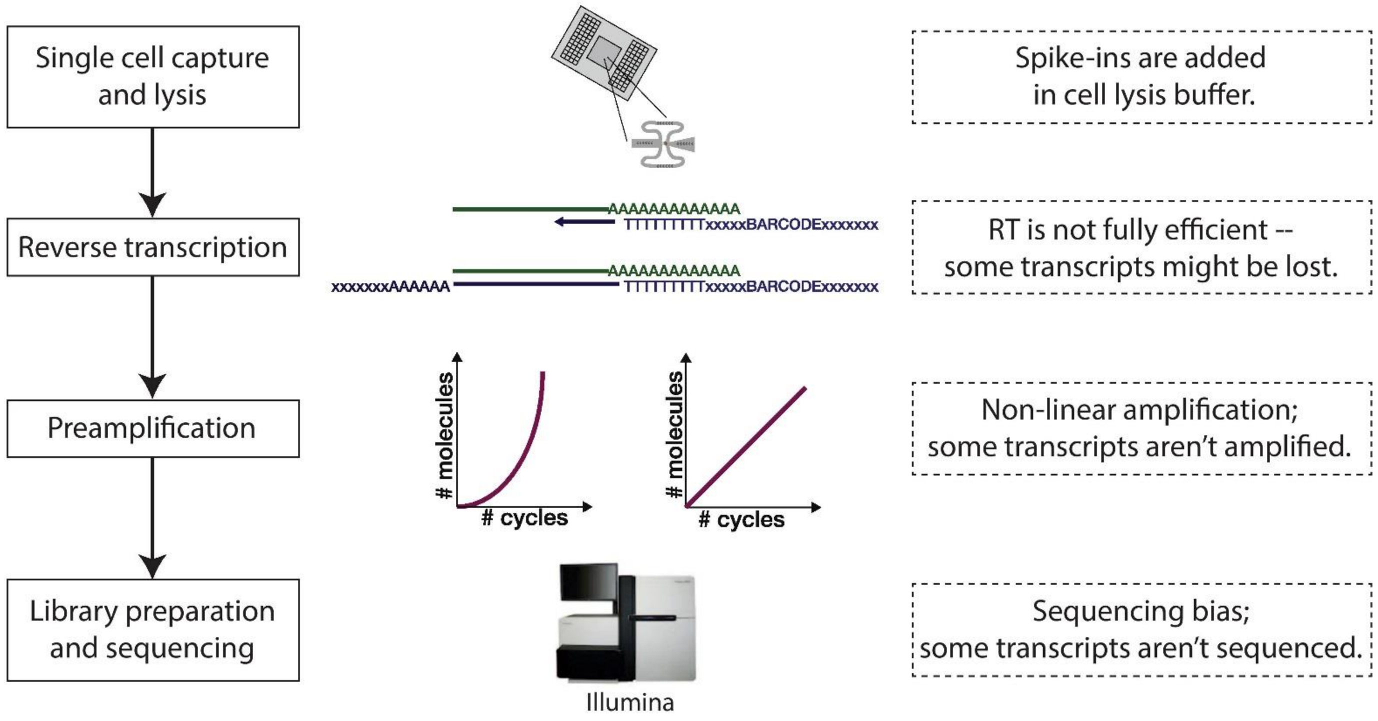

Figure S2. Cell size and cell cycle affects transcriptional bursting. Large cell size leads to large burst size due to *trans*-effect whereas cells with duplicated DNAs in G2 phase have decreased burst frequency due to *cis*-effect. Spike-ins are added as internal controls. Plot is partially adapted from Padovan-Merhar et al. [1].

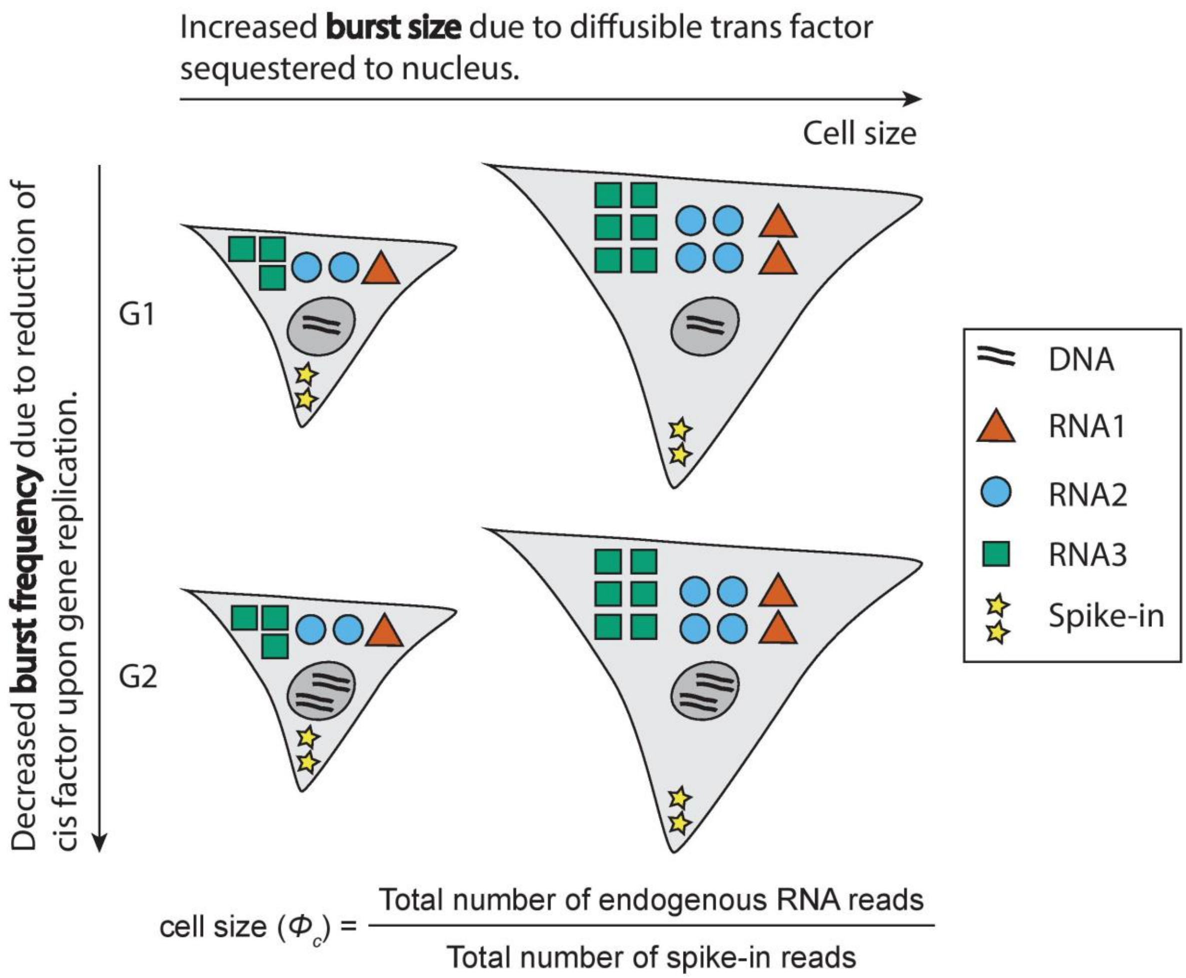

Figure S3. F1 cross mouse model to generate allele-specific read counts from scRNA-seq. Single cells from F1 hybrids generated between CAST female with C57 male mice permits detection of parental allele-specific expression at heterozygous loci. Plot is partially adapted from Ginart et al. [2].

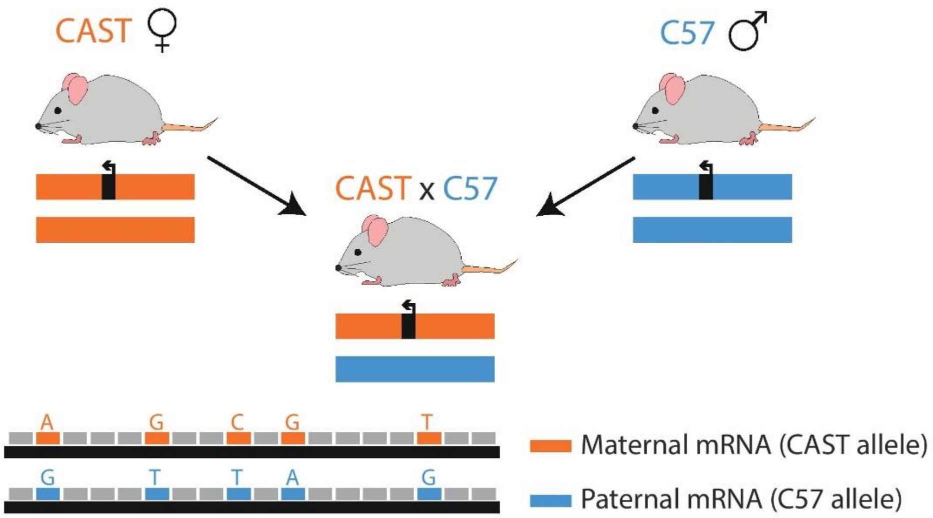

Figure S4. PCA analysis on maternal allelic proportion and RPKM. Cells from different stages are colored differently. Both the maternal allelic proportion and the RPKM are informative in separating different cell stages. While the first three PCs well separate the early-stage cells from blastocyst cells, the early, mid, and late blastocyst cluster are combined separately to infer kinetic parameters.

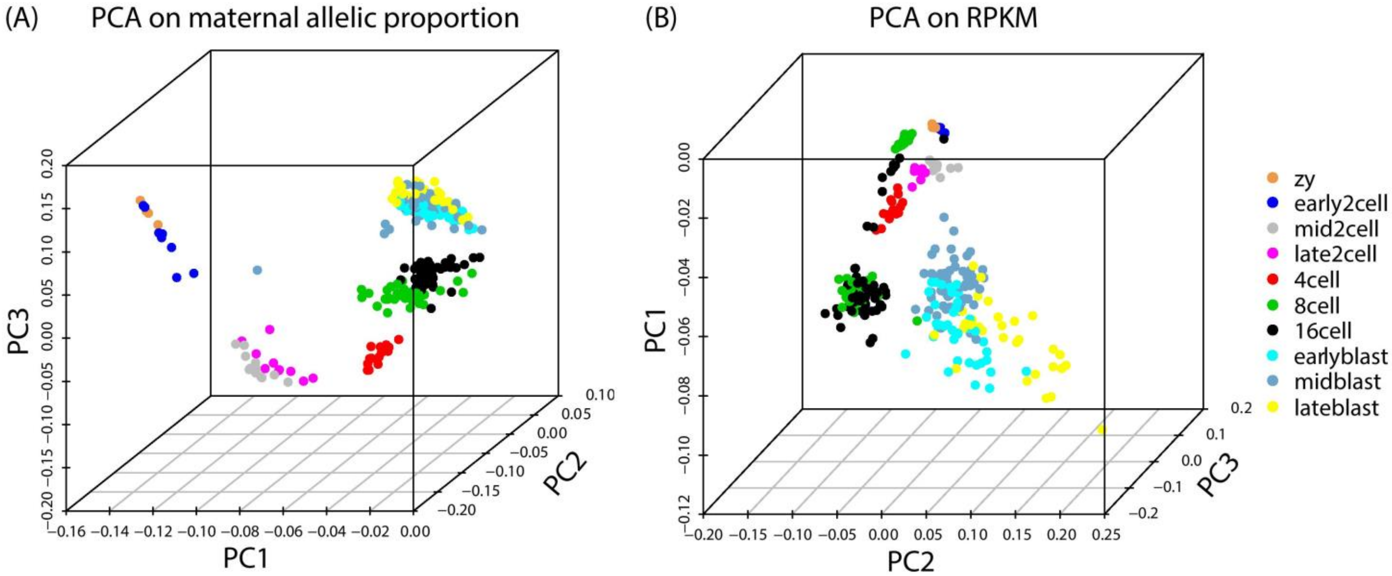

Figure S5. Modeling of technical variability and parameter estimation. Amplification and sequencing bias are modeled and captured by parameter α and β. Estimation is carried out by log-linear regression. Probability of dropout is modeled by κ and τ and depends on the logarithm of the true expression. Estimation is carried out by the Nelder-Mead simplex algorithm. (A) Estimation results from 8 spike-ins from mouse blastocyst cells [3]. The percentage of zero read counts are decomposed into those from Poisson sampling and those from dropout (spike-ins are non-bursty). (B) Estimation results from 92 ERCC spike-ins from human fibroblast cells [4].

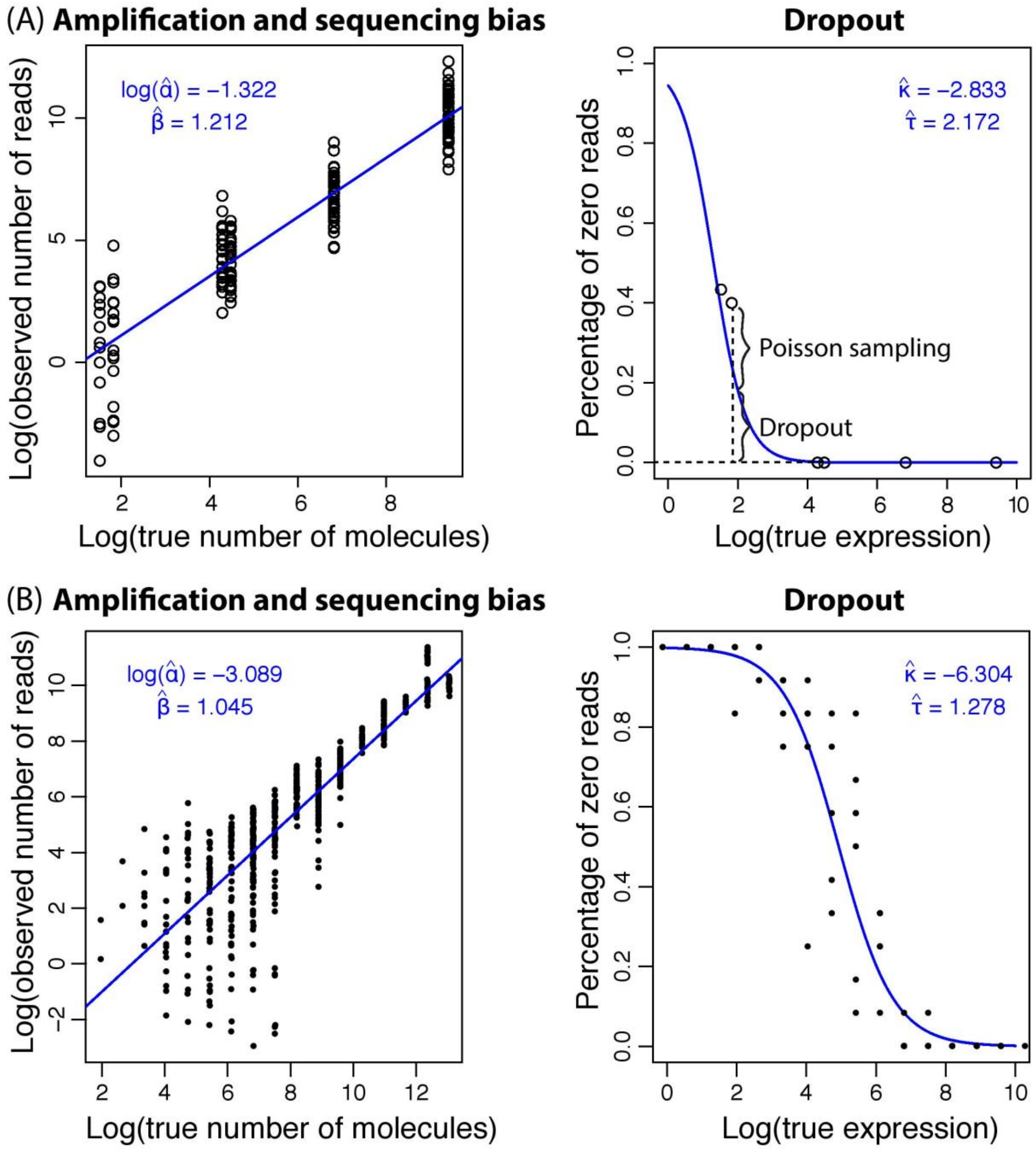

Figure S6. Gene categorization results on scRNA-seq dataset of mouse blastocyst and human fibroblast cells. For each gene, the proportion of cells expressing neither, one, or both alleles, estimated through the Bayes procedure are denoted as p_0, p_1, and p_2. The smoothed scatterplot of p_2 against p_0 across all genes is shown. If the two alleles of a gene are expressed in a coordinated fashion, then there is no monoallelic expression and thus $p_0 + p_2 = 1$, which corresponds to the diagonal line. If the two alleles fire independently and *share the same bursting kinetics*, let $p = p_A = p_B$ be the proportion of cells expressing each allele, then we have $p_0 = (1-p)^2$, $p_1 = 2p(1-p)$, and $p_2 = p^2$. This corresponds to the red curve, where $p_2 = \left(\sqrt{p_0} - 1\right)^2$. The observed data, on the genome-wide scale, generally don't show significant deviations from this red curve, providing visual evidence that for most genes the assumption of shared bursting kinetics and independent bursting between the two alleles is reasonable. Smooth scatterplot is plotted by smoothScatter function in R. For genes that are significantly deviated, hypothesis testing is carried out to determine whether it is due to differential bursting kinetics and/or non-independent bursting between the two alleles. (A) Results from mouse blastocyst cells. (B) Results from human fibroblast cells.

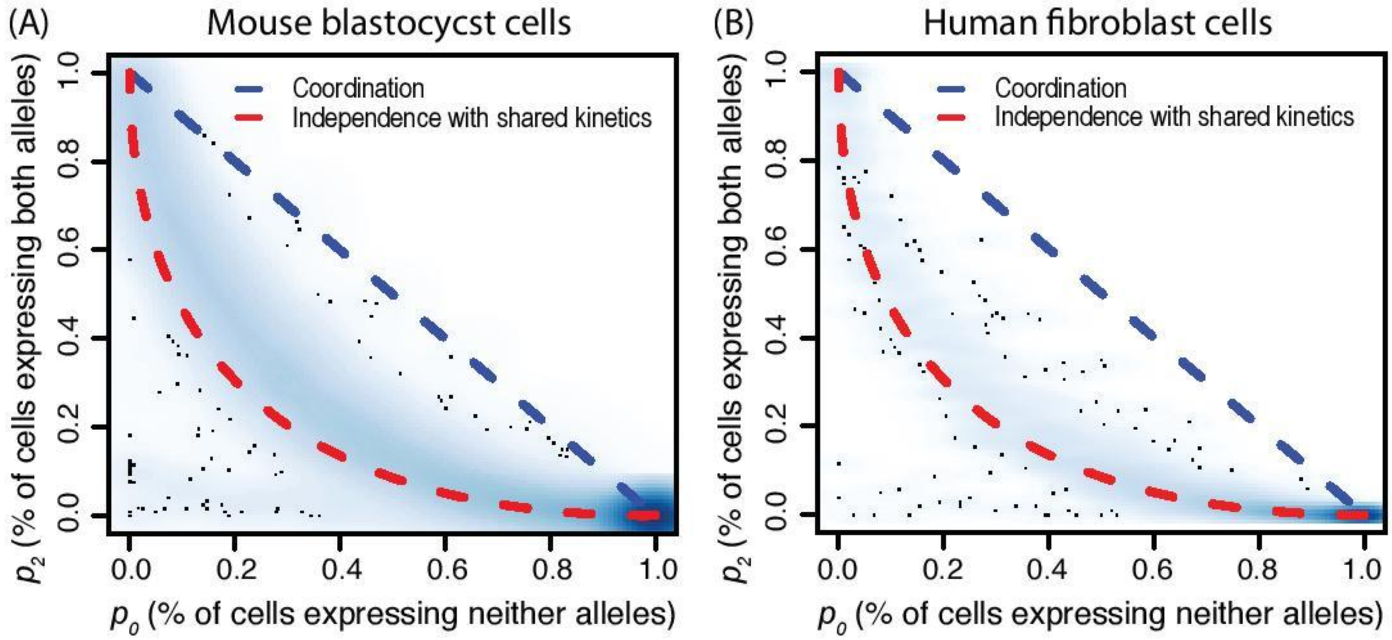

Figure S7. Histogram repiling method for kinetic parameter estimation with adjustment of technical variability. Histogram of number of cells with observed read counts Q is shown in light blue; histogram of number of cells with true number of molecules Y is shown in light red. Three example genes are plotted: (A) Partial cells with zero read counts of a bursty gene are due to dropout events and are recovered to non-zero true number of molecules; (B) Gene is off in most cells; (C) Gene is constitutively expressed with expression levels adjusted for sequencing and amplification bias.

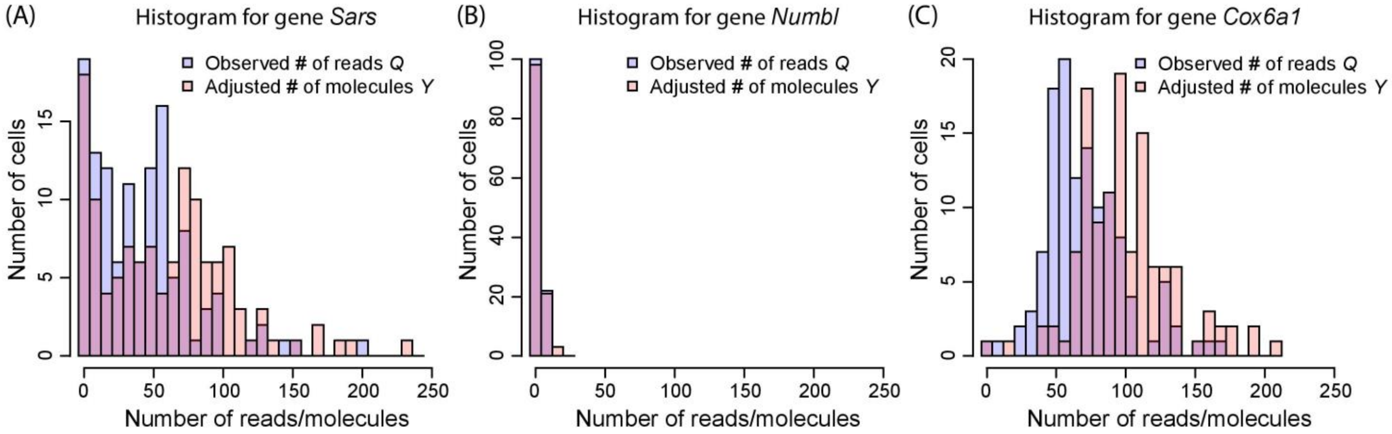

Figure S8. Allele-specific kinetic parameter estimation using bursty X-chromosome genes as positive controls. When the sample pool is mixed with male (X^AY) and female (X^AX^B) cells, the maternal A allele has significantly higher burst frequency than the paternal B allele while the burst size difference remains insignificant. When the sample pool consists of male (X^AY) cells only, the bursty X-chromosome genes are categorized as maternal monoallelic A expression, whose allelic kinetic parameters for the paternal B allele are not estimable. X-chromosome genes serve as a positive control and a sanity check, which shows that SCALE estimates the allele-specific kinetics as is expected.

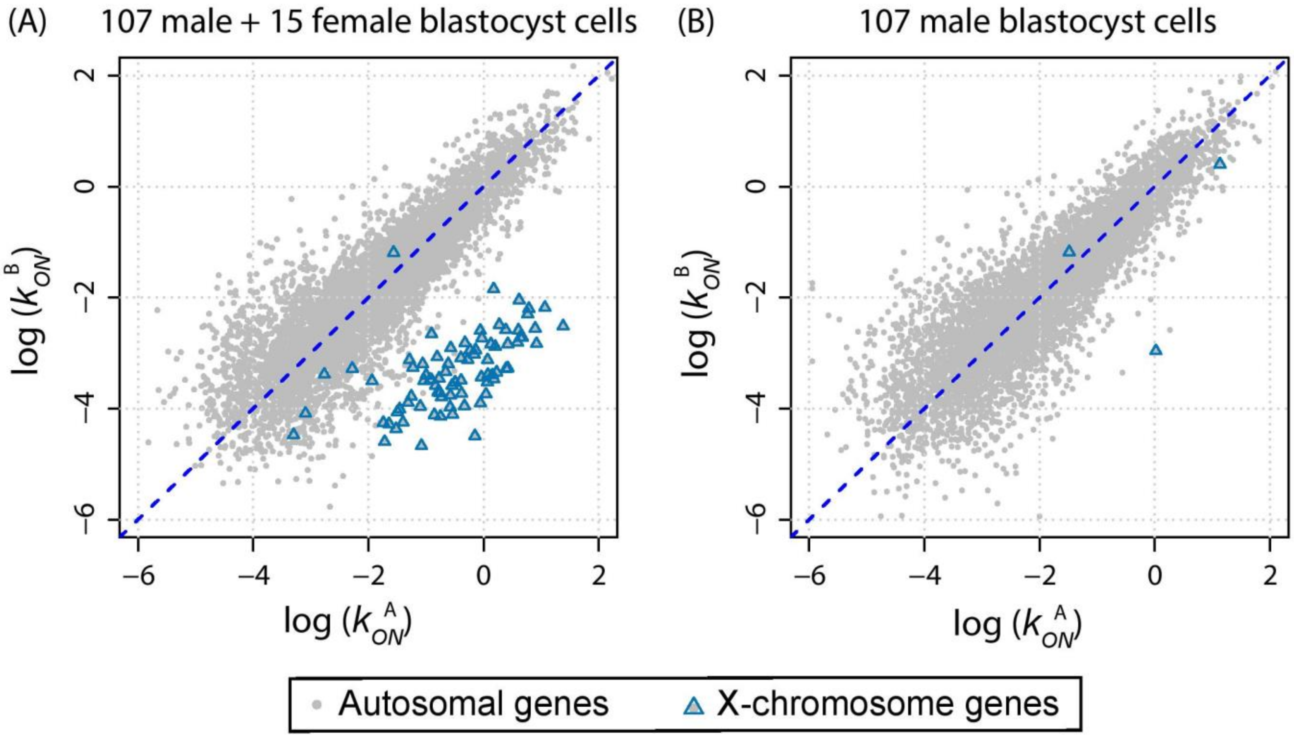

Figure S9. Correlation between bursting kinetics and expression levels. Pairwise correlation analysis is performed between burst frequency k_{on}^{A} and k_{on}^{B}, proportion of unit time that the gene stays in the active state $p^{A}=k_{on}^{A}/(k_{on}^{A}+k_{off}^{A})$ and $p^{B}=k_{on}^{B}/(k_{on}^{B}+k_{off}^{B})$, burst size s^{A}/k_{off}^{A} and s^{B}/k_{off}^{B}, and total number of reads across all cells $\sum_{c} Q_{c}^{A}$ and $\sum_{c} Q_{c}^{B}$. Each dot is one gene from the human fibroblast dataset. Expression levels are highly correlated with burst frequency as well as the fraction of time that the gene stays 'ON', but not with burst size.

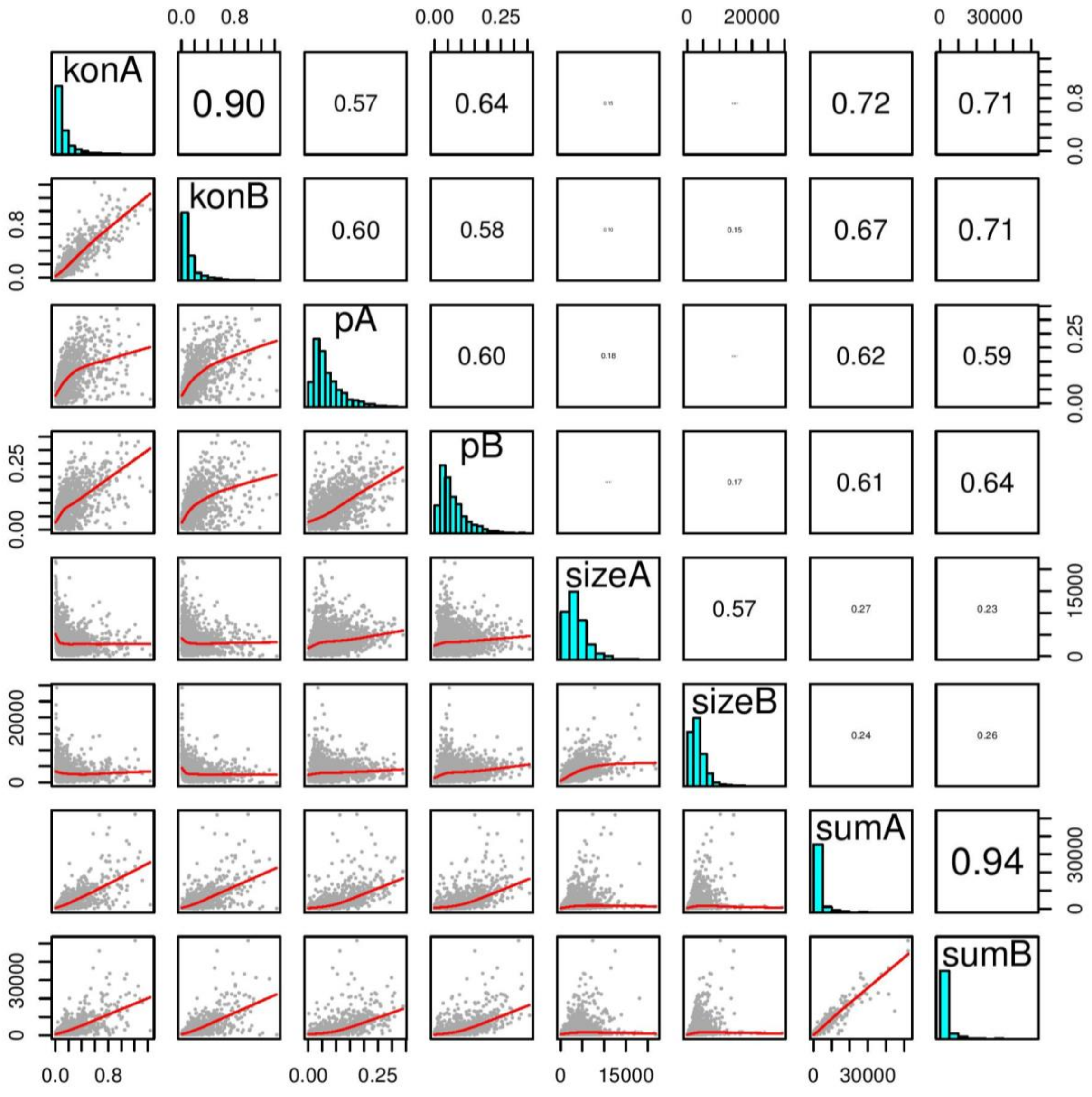

Figure S10. Three classes of Poisson-Beta transcription model. Each dot corresponds to a cell, whose read count is generated *in silico* with underlying true parameters shown in each panel. (A) Genes with small k_{on} and small k_{off} are bursty, whose bursting kinetic parameters are identifiable. (B) Genes with large k_{on} and small k_{off} are typically highly expressed – the system collapses down to a constitutive expression model, resulting in a Poisson or negative-Binomial-like distribution. (C) Genes with small k_{on} and large k_{off} have low expression in most cells and high expression in a small number of cells, shown as a long exponential tail. (D) Genes with large k_{on} and large k_{off} are statistically hard to be distinguished from genes shown in (B).

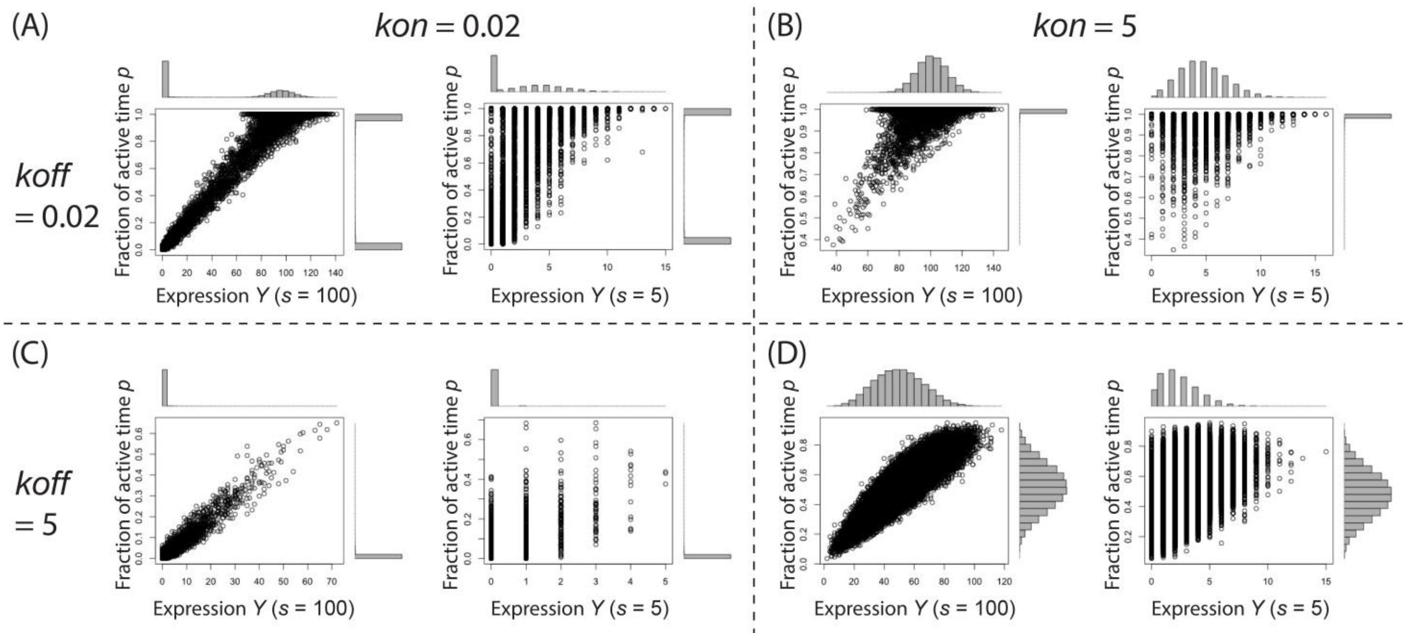

Figure S11. Assessment of moment estimators by simulations studies. Estimation accuracy is measured by relative estimation error $|\hat{\theta}-\theta|/\theta$ for k_{on}, k_{off}, s, and s/k_{off}. Simulation is carried out with different underlying true parameters across 100 and 1000 cells: (A) varied k_{on} with fixed s and k_{off} acorss 100 cells; (B) varied k_{off} with fixed s and k_{on} across 100 cells; (C) varied s with fixed k_{on} and k_{off} across 100 cells; (D) varied k_{on} with fixed s and k_{off} acorss 1000 cells; (E) varied k_{off} with fixed s and k_{on} across 1000 cells; (F) varied s with fixed k_{on} and k_{off} across 1000 cells. Cases where $k_{on} \ll k_{off}$ (silence) and $k_{on} \gg k_{off}$ (constitutive expression), shown as red and black curves, have high estimation errors. k_{off} has higher estimation uncertainty than s in burst size.

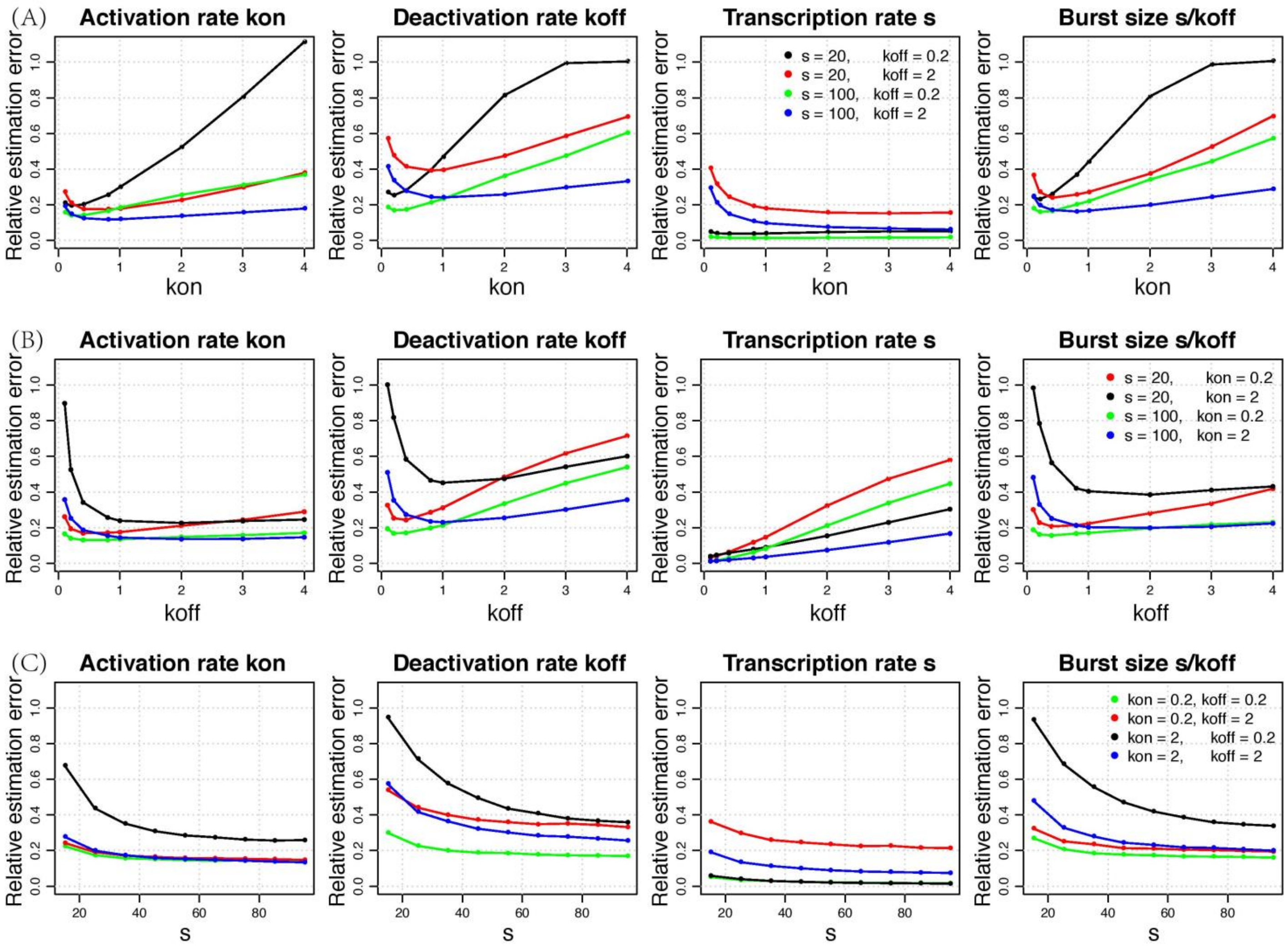

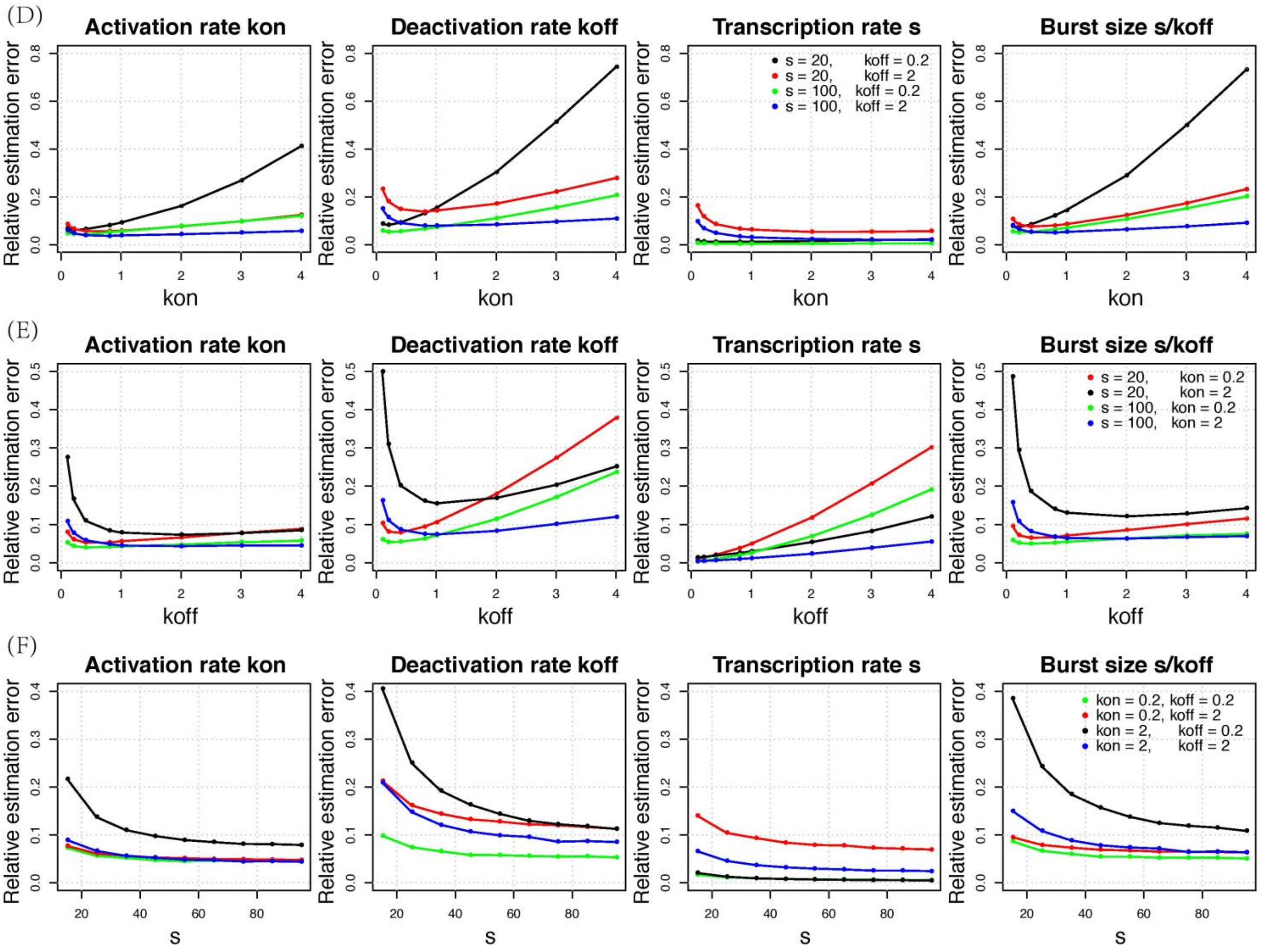
(D)
Activation rate kon
Deactivation rate koff
Transcription rate s
Burst size s/koff
Relative estimation error
kon
s = 20, koff = 0.2
s = 20, koff = 2
s = 100, koff = 0.2
s = 100, koff = 2
(E)
Activation rate kon
Deactivation rate koff
Transcription rate s
Burst size s/koff
Relative estimation error
koff
s = 20, kon = 0.2
s = 20, kon = 2
s = 100, kon = 0.2
s = 100, kon = 2
(F)
Activation rate kon
Deactivation rate koff
Transcription rate s
Burst size s/koff
Relative estimation error
s
kon = 0.2, koff = 0.2
kon = 0.2, koff = 2
kon = 2, koff = 0.2
kon = 2, koff = 2

Figure S12. Correlation between allelic burst size s/k_{off}, transcription rate s, and deactivation rate k_{off}. Over/under estimation of s is compensated by over/under estimation of k_{off}, resulting in the ratio burst size (s/k_{off}) having higher correlation between the two alleles. Each point is a biallelic bursty gene, whose kinetic parameters are estimated from real dataset of (A) 122 mouse blastocyst cells [3] and (B) 104 human fibroblast cells [4].

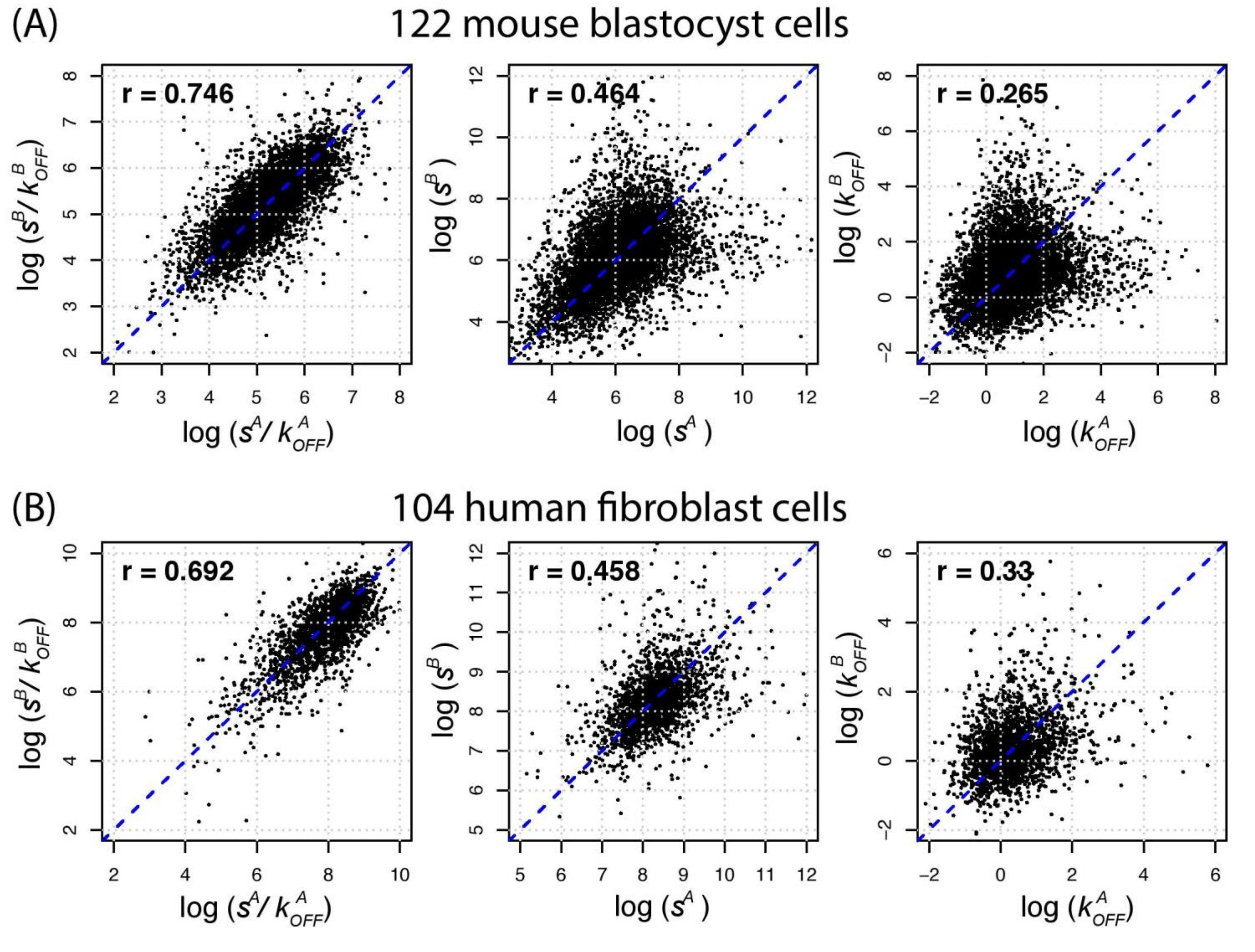

Figure S13. Power analysis for hypothesis testing of differential burst frequency and burst size between the two alleles. The null hypothesis is both alleles sharing the same bursting kinetics ($k_{on}^A = k_{on}^B = 0.2, k_{off}^A = k_{off}^B = 0.2, s^A = s^B = 50$). Different alternative hypotheses are included in the figure legends: (A) differential burst frequency; (B) differential burst size due to change in s; and (C) differential burst size due to change in k_{off}. Overall, the testing of burst frequency and burst size have similar power with relatively low power if the allelic difference in burst size is due to difference in the deactivation rate k_{off}. Power is evaluated at 0.05 significance level, suggesting a reduced power if a more stringent p-value cutoff is adopted.

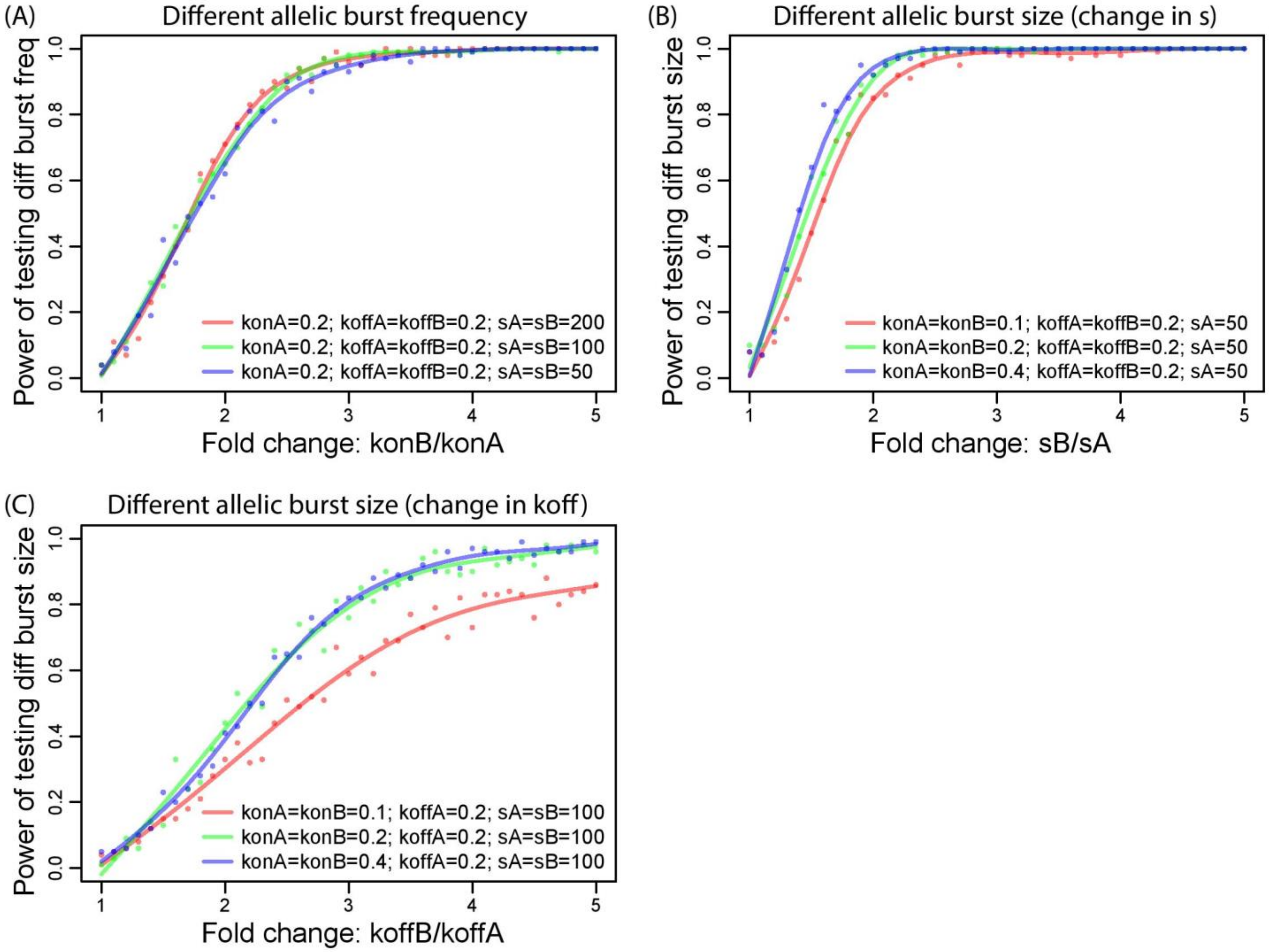

Figure S14. Adjustment of cell size and technical variability leads to more accurate estimation of allelic bursting kinetics. Relative estimation error of burst frequency and burst size are measured through 5000 simulations across 100 and 400 cells respectively with fixed underlying true allele-specific kinetics ($k_{on}^{A} = k_{on}^{B} = k_{off}^{A} = k_{off}^{B} = 0.2, s^{A} = s^{B} = 100$). Technical variability is simulated with the estimated parameters from the mouse blastocyst dataset (Figure S5A). Cell size is simulated from a normal distribution with mean 1 and standard deviation 0.1 and 0.01 respectively. SCALE is applied in its default setting, without accounting for cell size, without adjustment of technical variability, and not in an allele-specific manner (using total coverage as input). SCALE in its default setting has the smallest relative estimation error across all four parallel runs. The estimation accuracy improves as the number of cells increases.

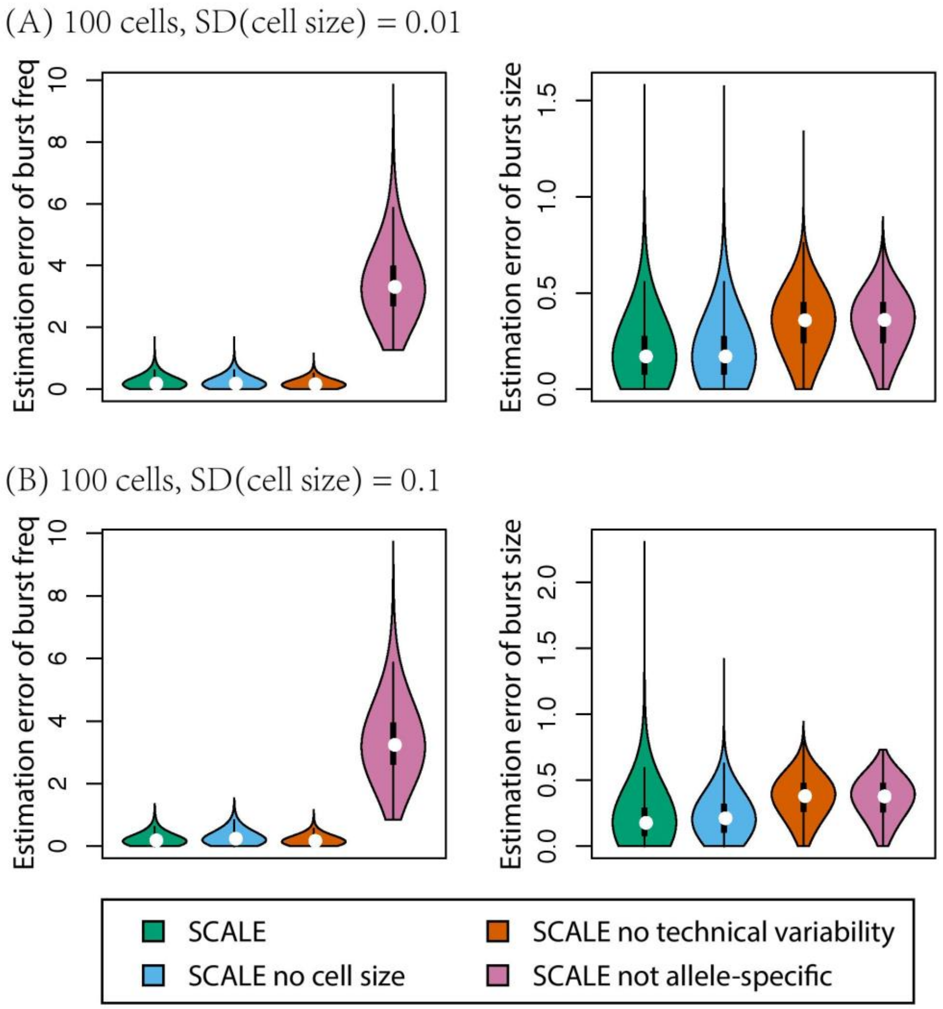

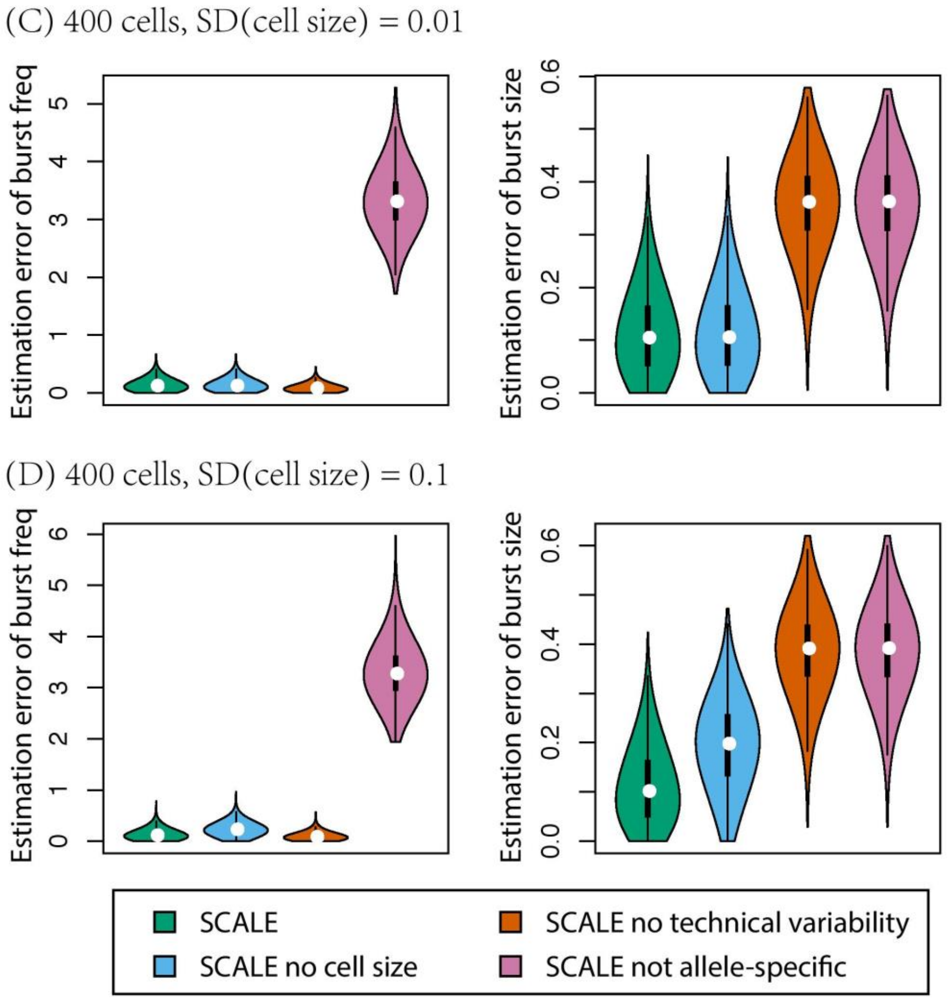
(C) 400 cells, SD(cell size) = 0.01
Estimation error of burst freq
Estimation error of burst size
(D) 400 cells, SD(cell size) = 0.1
Estimation error of burst freq
Estimation error of burst size
SCALE
SCALE no cell size
SCALE no technical variability
SCALE not allele-specific

Figure S15. Adjustment of cell size and technical variability leads to more accurate estimation of allelic bursting kinetics. Relative estimation error of burst frequency and burst size are measured through 5000 simulations across 100 and 400 cells respectively with fixed underlying true allele-specific kinetics ($k_{on}^{A} = k_{on}^{B} = k_{off}^{A} = k_{off}^{B} = 0.2, s^{A} = s^{B} = 100$). Technical variability is simulated with the estimated parameters from the human fibroblast dataset (Figure S5B). Cell size is simulated from a normal distribution with mean 1 and standard deviation 0.1 and 0.01 respectively. SCALE is applied in its default setting, without accounting for cell size, without adjustment of technical variability, and not in an allele-specific manner (using total coverage as input). SCALE in its default setting has the smallest relative estimation error across all four parallel runs. The estimation accuracy improves as the number of cells increases. Logarithm of the estimation error is shown as the Y-axis due to the completely-off estimation using total instead of allele-specific expression.

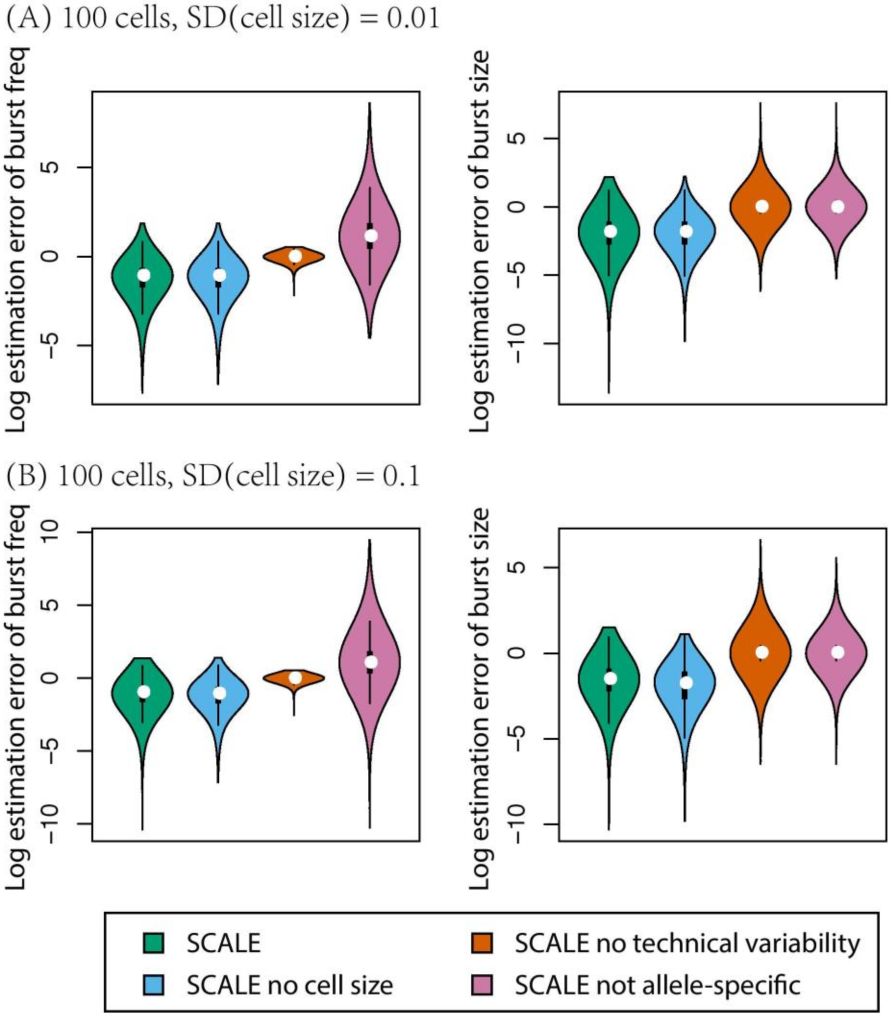

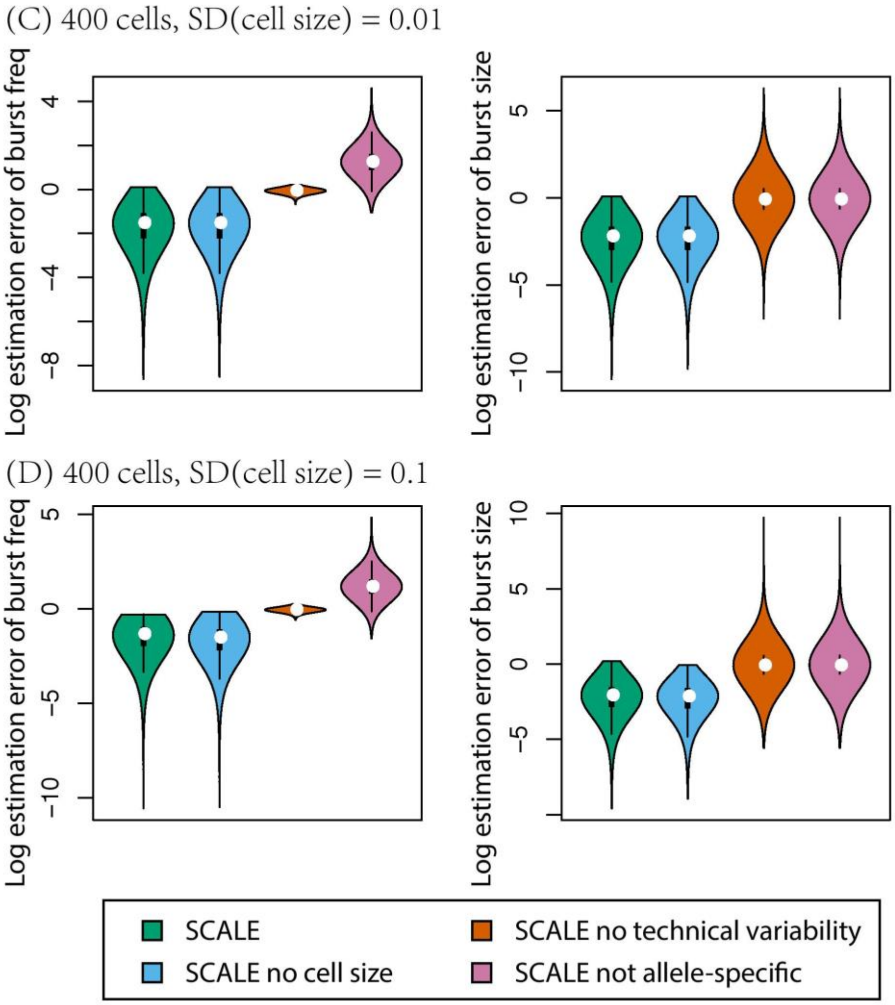
(C) 400 cells, SD(cell size) = 0.01
Log estimation error of burst freq
Log estimation error of burst size
(D) 400 cells, SD(cell size) = 0.1
Log estimation error of burst freq
Log estimation error of burst size
SCALE
SCALE no cell size
SCALE no technical variability
SCALE not allele-specific

Table S1-S4. Spike-in input and SCALE output for mouse blastocyst and human fibroblast scRNA-seq data. True number of molecules, lengths, as well as observed number of reads for each spike-in RNA across all cells are used as input to model technical variability in (Additional file 2: Table S1) 122 mouse blastocyst cells and (Additional file 4: Table S3) 104 human fibroblast cells. SCALE output includes the inferred allelic bursting kinetics as well as the hypothesis testing results at heterozygous loci across all genes from (Additional file 3: Table S2) the mouse blastocyst dataset and (Additional file 5: Table S4) the human fibroblast dataset. The table is separately attached as an excel file. Separately attached as Additional file 2-5.

Table S5. Standard errors and confidence intervals of estimated kinetic parameters. Simulated dataset are generated from the Poisson-Beta transcriptional model with true underlying parameters shown in first row. Bootstrap resampling gives standard errors and confidence intervals of the moment estimates. Standard errors are large with unstable moment estimates for genes with $k_{on} \ll k_{off}$ (silence) and $k_{on} \gg k_{off}$ (constitutive expression).

**Classes**		Small k_{on} Small k_{off}	Small k_{on} Large k_{off}	Large k_{on} Small k_{off}	Large k_{on} Large k_{off}
**True value**	k_{on}	0.02	0.02	2	2
	k_{off}	0.02	2	0.02	2
	s	100	100	100	100
**Estimate Standard error Confidence Interval**	k_{on}	0.021<br>0.013<br>(-0.005, 0.047)	0.023<br>0.007<br>(0.01, 0.036)	1.973<br>616.977<br>(-17.216, 19.312)	1.964<br>0.324<br>(1.46, 2.721)
	k_{off}	0.022<br>0.015<br>(-0.005, 0.053)	1.662<br>13.185<br>(0.328, 5.51)	0.024<br>635.842<br>(0, 2.804)	2.13<br>0.921<br>(1.136, 4.546)
	s	99.1<br>1.537<br>(95.98, 102.01)	98.43<br>362.895<br>(24.48, 198.69)	100.49<br>30.995<br>(96.2, 105.03)	102.23<br>11.944<br>(86.48, 131.9)

Supplementary Methods

Bioinformatic pipeline: calling heterozygous loci within coding regions from bulk-tissue sequencing (also available via GitHub)

```
# 1. Index the genome template
bwa index ~/structure/hg19/ucsc.hg19.fasta

# 2. Align reads in .fastq file to the template (sequenced at two different lanes)
bwa mem -M -t 16 ~/structure/hg19/ucsc.hg19.fasta
_EGAR00001248897_UCF_1014_NoIndex_L008_R1_001.fastq
_EGAR00001248897_UCF_1014_NoIndex_L008_R2_001.fastq > _EGAR00001248897_UCF_1014_NoIndex_L008.sam

bwa mem -M -t 16 ~/structure/hg19/ucsc.hg19.fasta
_EGAR00001248899_UCF1014_NoIndex_L002_R1_001.fastq
_EGAR00001248899_UCF1014_NoIndex_L002_R2_001.fastq > _EGAR00001248899_UCF1014_NoIndex_L002.sam

# 3. Convert .sam to .bam and sort
samtools view -bS _EGAR00001248897_UCF_1014_NoIndex_L008.sam >
_EGAR00001248897_UCF_1014_NoIndex_L008.bam
line=_EGAR00001248897_UCF_1014_NoIndex_L008.bam
java -jar ~/bin/SortSam.jar INPUT=$line OUTPUT=$line.sorted.bam SORT_ORDER=coordinate

samtools view -bS _EGAR00001248899_UCF1014_NoIndex_L002.sam >
_EGAR00001248899_UCF1014_NoIndex_L002.bam
line=_EGAR00001248899_UCF1014_NoIndex_L002.bam
java -jar ~/bin/SortSam.jar INPUT=$line OUTPUT=$line.sorted.bam SORT_ORDER=coordinate

# 4. Add read group
line=_EGAR00001248897_UCF_1014_NoIndex_L008.bam
#line=_EGAR00001248899_UCF1014_NoIndex_L002.bam

java -jar ~/bin/AddOrReplaceReadGroups.jar INPUT=$line.sorted.bam OUTPUT=$line.sorted.rg.bam
RGID=$line RGLB=WGS_UCF1014 RGPL=ILLUMINA RGPU=machine RGSM=UCF1014
samtools index $line.sorted.rg.bam

# 5. Merge two different lanes
samtools merge UCF1014.merge.bam _EGAR00001248899_UCF1014_NoIndex_L002.bam.sorted.rg.bam
_EGAR00001248897_UCF_1014_NoIndex_L008.bam.sorted.rg.bam
java -jar ~/bin/SortSam.jar INPUT=UCF1014.merge.bam OUTPUT=UCF1014.merge.sorted.bam
SORT_ORDER=coordinate
samtools index UCF1014.merge.sorted.bam

# 6. Dedup
java -jar ~/bin/MarkDuplicates.jar INPUT=UCF1014.merge.sorted.bam
OUTPUT=UCF1014.merge.sorted.dedup.bam  METRICS_FILE=UCF1014.merge.sorted.dedup.metrics.txt
PROGRAM_RECORD_ID= MarkDuplicates PROGRAM_GROUP_VERSION=null PROGRAM_GROUP_NAME=MarkDuplicates
java -jar ~/bin/BuildBamIndex.jar INPUT=UCF1014.merge.sorted.dedup.bam

# 7. Realign
java -jar ~/bin/GenomeAnalysisTK.jar -T RealignerTargetCreator -R
/home/stat/yuchaoj/structure/hg19/ucsc.hg19.fasta -I UCF1014.merge.sorted.dedup.bam -known
/home/stat/yuchaoj/structure/hg19/Mills_and_1000G_gold_standard.indels.hg19.sites.vcf -known
/home/stat/yuchaoj/structure/hg19/1000G_phase1.indels.hg19.sites.vcf -o
UCF1014.merge.sorted.dedup.target_intervals.list
```

```
java -jar ~/bin/GenomeAnalysisTK.jar -T IndelRealigner -R
/home/stat/yuchaoj/structure/hg19/ucsc.hg19.fasta -I UCF1014.merge.sorted.dedup.bam -
targetIntervals UCF1014.merge.sorted.dedup.target_intervals.list -known
/home/stat/yuchaoj/structure/hg19/Mills_and_1000G_gold_standard.indels.hg19.sites.vcf -known
/home/stat/yuchaoj/structure/hg19/1000G_phase1.indels.hg19.sites.vcf -o
UCF1014.merge.sorted.dedup.realigned.bam

# 8. Recalibrate
java -jar ~/bin/GenomeAnalysisTK.jar -T BaseRecalibrator -R
/home/stat/yuchaoj/structure/hg19/ucsc.hg19.fasta -I UCF1014.merge.sorted.dedup.realigned.bam -
knownSites /home/stat/yuchaoj/structure/hg19/dbsnp_138.hg19.vcf -knownSites
/home/stat/yuchaoj/structure/hg19/Mills_and_1000G_gold_standard.indels.hg19.sites.vcf -knownSites
/home/stat/yuchaoj/structure/hg19/1000G_phase1.indels.hg19.sites.vcf -o
UCF1014.merge.sorted.dedup.realigned.recal_data.table

java -jar ~/bin/GenomeAnalysisTK.jar -T PrintReads -R
/home/stat/yuchaoj/structure/hg19/ucsc.hg19.fasta -I UCF1014.merge.sorted.dedup.realigned.bam -
BQSR UCF1014.merge.sorted.dedup.realigned.recal_data.table -o
UCF1014.merge.sorted.dedup.realigned.recal.bam
samtools index UCF1014.merge.sorted.dedup.realigned.recal.bam

# 9. GATK HaplotypeCaller
java -jar ~/bin/GenomeAnalysisTK.jar -R ~/structure/hg19/ucsc.hg19.fasta -T HaplotypeCaller -I
UCF1014.merge.sorted.dedup.realigned.recal.bam -o
UCF1014.merge.sorted.dedup.realigned.recal.raw.snps.indels.g.vcf
```

The above SNP calling procedure by GATK HaplotypeCaller altogether returned 4,555,158 loci from the whole-genome sequencing of female human fibroblast cells [4]. Adopted QC procedure below to filter out calls.

**QC procedure**	**Number of calls**
GATK HaplotypeCaller	4,555,158
Heterozygous loci (genotype 0/1)	2,857,194
Point mutations, not indels	2,424,074
Exonic + UTR3 + UTR5 regions (ANNOVAR*)	32,117
Not within segmental duplication regions	28,596
Reported in the 1000 Genomes Project	26,543
Unique genes+	**9,016**

* ANNOVAR annotations: 58.8% from intergenic regions, 32.5% from intronic regions, 1.3% from exonic, UTR3, and UTR5 regions.

```
table_annovar.pl 1_annovar_input.txt /home/stat/yuchaoj/yuchaoj/program/annovar/humandb/ -buildver
hg19 -out 1_UCF1014_annovar_output_ -remove -protocol
refGene,cytoBand,genomicSuperDups,esp6500siv2_all,1000g2014oct_all,1000g2014oct_afr,1000g2014oct_e
as,1000g2014oct_eur,ljb26_all -operation g,r,r,f,f,f,f,f,f -nastring .
```

+ If multiple heterozygous loci exist within the same gene, select the one with the highest coverage within exonic regions (if there are only calls within UTR, use UTR calls).

Bioinformatic pipeline: getting allelic read counts at heterozygous loci from scRNA-seq (also available via GitHub)

```
# 0. Get splice junction database:
wget http://labshare.cshl.edu/shares/gingeraslab/www-
data/dobin/STAR/STARgenomes/GENCODE/Old/gencode.v14.annotation.gtf.sjdb

# 1. Generate the genome using STAR, 100bp PE
genomeDir=/home/yuchaoj/hg19
STAR --runMode genomeGenerate --genomeDir $genomeDir --genomeFastaFiles ~/hg19/hg19.fa --
sjdbFileChrStartEnd ~/hg19/gencode.v14.annotation.gtf.sjdb --sjdbOverhang 99 --runThreadN 4

# 2. Alignment jobs were executed as follows
genomeDir=/home/yuchaoj/hg19
while read samp
do
echo 'STAR --genomeDir '$genomeDir' --readFilesIn '''$samp'''_1.fastq '''$samp'''_2.fastq --
outFilterIntronMotifs RemoveNoncanonicalUnannotated --outFileNamePrefix '$samp'_ --runThreadN 4' |
bsub -M 60000
done < samp.list

# 3. Convert .sam to .bam
while read samp; do
echo 'samtools view -bS '''$samp'''_Aligned.out.sam > '''$samp'''_Aligned.out.bam ' | bsub -M
60000
done < samp.list

# 4. Filter & sort
while read samp; do
echo 'perl filter_sam_v2.pl '''$samp'''_Aligned.out.bam '''$samp'''_Aligned.out.filtered.sam;
samtools view -bS '''$samp'''_Aligned.out.filtered.sam > '''$samp'''_Aligned.out.filtered.bam' |
bsub -M 60000
done < samp.list

while read samp; do
echo 'java -Xmx30G -jar ~/bin/SortSam.jar INPUT='''$samp'''_Aligned.out.filtered.bam
OUTPUT='''$samp'''_Aligned.out.filtered.sorted.bam SORT_ORDER=coordinate ' | bsub -M 40000
done < samp.list

# 5. Add read group and index
while read samp; do
echo 'java -Xmx30G -jar ~/bin/AddOrReplaceReadGroups.jar
INPUT='''$samp'''_Aligned.out.filtered.sorted.bam
OUTPUT='''$samp'''_Aligned.out.filtered.sorted.rg.bam RGID=UCF1014 RGLB=scRNA_seq_UCF1014
RGPL=ILLUMINA RGPU=machine RGSM='$samp'; samtools index
'''$samp'''_Aligned.out.filtered.sorted.rg.bam'  | bsub -M 40000
done < samp.list

# 6. Parse file: position.txt contains all the heterozygous loci (chr + coordinate) returned by
GATK HaplotypeCaller using WGS.
while read bam; do
echo ' samtools mpileup -E -f /home/yuchaoj/hg19/hg19.fa -d 1000000 --position position.txt '$bam'
> '$bam'.mpileup;
perl pileup2base_no_strand.pl '$bam'.mpileup 30 '$bam'.parse30.txt
' | bsub -M 20000
done < rg.bam.list
```

Bioinformatic pipeline: getting number of reads for spike-ins from scRNA-seq (also available via GitHub)

```
# 0. concatenate ERCC with hg19 and index the fasta file
cat ERCC92.fa hg19.fa > hg19_ERCC.fa
java -jar ~/bin/CreateSequenceDictionary.jar R= hg19_ERCC.fa O= hg19_ERCC.dict
samtools faidx hg19_ERCC.fa

# 1. Generate the genome using STAR, 50bp PE
genomeDir=/home/yuchaoj/hg19_ERCC
STAR --runMode genomeGenerate --genomeDir $genomeDir --genomeFastaFiles ~/hg19_ERCC/hg19_ERCC.fa -
-sjdbFileChrStartEnd ~/hg19/gencode.v14.annotation.gtf.sjdb --sjdbOverhang 49 --runThreadN 4

# 2. Alignment jobs were executed as follows
genomeDir=/home/yuchaoj/hg19_ERCC
while read samp; do
echo 'STAR --genomeDir '$genomeDir' --readFilesIn '''$samp'''_R1_001.fastq
'''$samp'''_R2_001.fastq --outFilterIntronMotifs RemoveNoncanonicalUnannotated --outFileNamePrefix
'$samp'_ --runThreadN 4' | bsub -M 60000
done < samp_ERCC.list

# 3. Convert .sam to .bam
while read samp; do
echo 'samtools view -bS '''$samp'''_Aligned.out.sam > '''$samp'''_Aligned.out.bam ' | bsub -M
60000
done < samp_ERCC.list

# 4. Filter & Sort
while read samp; do
echo 'perl filter_sam_v2.pl '''$samp'''_Aligned.out.bam '''$samp'''_Aligned.out.filtered.sam;
samtools view -bS '''$samp'''_Aligned.out.filtered.sam > '''$samp'''_Aligned.out.filtered.bam' |
bsub -M 60000
done < samp_ERCC.list

while read samp; do
echo 'java -Xmx30G -jar ~/bin/SortSam.jar INPUT='''$samp'''_Aligned.out.filtered.bam
OUTPUT='''$samp'''_Aligned.out.filtered.sorted.bam SORT_ORDER=coordinate ' | bsub -M 40000
done < samp_ERCC.list

# 5. Add read group and index
while read samp; do
echo 'java -Xmx30G -jar ~/bin/AddOrReplaceReadGroups.jar
INPUT='''$samp'''_Aligned.out.filtered.sorted.bam
OUTPUT='''$samp'''_Aligned.out.filtered.sorted.rg.bam RGID=T2N_ERCC RGLB=scRNA_seq_T2N_ERCC
RGPL=ILLUMINA RGPU=machine RGSM='$samp'; samtools index
'''$samp'''_Aligned.out.filtered.sorted.rg.bam'  | bsub -M 30000
done < samp_ERCC.list

# 6. Get total read counts as well as read counts for ERCC
while read bam; do
echo $bam
samtools view -c $bam
done < rg.bam.list

while read ercc; do
echo $ercc
while read bam; do samtools view -c $bam $ercc;done < rg.bam.list | cat > $ercc.txt
done < ercc.id
```

References

1. Padovan-Merhar O, Nair GP, Biaesch AG, Mayer A, Scarfone S, Foley SW, Wu AR, Churchman LS, Singh A, Raj A: **Single mammalian cells compensate for differences in cellular volume and DNA copy number through independent global transcriptional mechanisms.** *Mol Cell* 2015, **58:**339-352.
2. Ginart P, Kalish JM, Jiang CL, Yu AC, Bartolomei MS, Raj A: **Visualizing allele-specific expression in single cells reveals epigenetic mosaicism in an H19 loss-of-imprinting mutant.** *Genes Dev* 2016, **30:**567-578.
3. Deng Q, Ramskold D, Reinius B, Sandberg R: **Single-cell RNA-seq reveals dynamic, random monoallelic gene expression in mammalian cells.** *Science* 2014, **343:**193-196.
4. Borel C, Ferreira PG, Santoni F, Delaneau O, Fort A, Popadin KY, Garieri M, Falconnet E, Ribaux P, Guipponi M, et al: **Biased allelic expression in human primary fibroblast single cells.** *Am J Hum Genet* 2015, **96:**70-80.

SCALE vignette

Yuchao Jiang
yuchaoj@upenn.edu

February 9, 2017

This is a demo for using the SCALE package in R. SCALE is a statistical framework for single cell allelic expression analysis. SCALE estimates kinetic parameters that characterize the transcriptional bursting process at the allelic level, while accounting for technical bias and other complicating factors such as cell size. SCALE detects genes with significantly different bursting kinetics between the two alleles, as well as genes where the two alleles exhibit dependence in their bursting processes.

SCALE's **webpage** is here. A **demo code** can be found here. Online **Q&A forum** for SCALE is available here. If you've any questions regarding the software, you can also email us at SCALE_scRNAseq@googlegroups.com.

1. Installation

R package SCALE is availble from GitHub ((https://github.com/yuchaojiang/SCALE):

```
> install.packages("devtools")
> library(devtools)
> install_github("yuchaojiang/SCALE/package")
```

2. SCALE workflow

2.1 Data input

The input to SCALE includes allele-specific read counts at heterozygous loci from single-cell RNA sequencing. The cells should be of the same cell types from the same tissue (i.e., they are homogeneous). Cell-wise quality control procedures based on sequencing depths, mean and standard deviation of allelic ratios are recommended. To control for technical variability, SCALE uses spike-ins. The spike-in input should be a matrix, where the rows correspond to spike-ins, the first column stores the true number of molecules, the second column stores the lengths of the spike-in molecules, and the third column and on store the observed read counts in each cell.

Below is a single-cell RNA sequencing dataset of 122 mouse blastocyst cells from Deng et al. (Science 2014), followed by step-by-step analysis breakdowns.

```
> library(SCALE)
> data(mouse.blastocyst)
> alleleA = mouse.blastocyst$alleleA # Read counts for A allele
> alleleB = mouse.blastocyst$alleleB # Read counts for B allele
> spikein_input = mouse.blastocyst$spikein_input # Spike-in input
> genename = rownames(alleleA)
```

```
> sampname = colnames(alleleA)
> head(colnames(alleleA))

[1] "GSM1112611" "GSM1112612" "GSM1112613" "GSM1112614" "GSM1112615"
[6] "GSM1112616"

> head(rownames(alleleA))

[1] "Hvcn1"  "Gbp7"   "Arrdc1" "Ercc5"  "Mrpl15" "Dclk1"

> rownames(spikein_input)

[1] "RNA_SPIKE_1"  "RNA_SPIKE_2"  "RNA_SPIKE_3"  "RNA_SPIKE_4"  "RNA_SPIKE_5A"
[6] "RNA_SPIKE_6"  "RNA_SPIKE_7"  "RNA_SPIKE_8"

> head(colnames(spikein_input))

[1] "spikein_mol"    "spikein_length" "GSM1112664"     "GSM1112665"
[5] "GSM1112666"     "GSM1112667"
```

2.2 Technical variability

A hierarchical model based on TASC (Toolkit for Analysis of Single Cell data) is fit to the spike-in data. Parameters $\{\alpha, \beta, \kappa, \tau\}$ associated with dropouts, amplification and sequencing bias are returned. A pdf plot is generated by default.

```
> abkt = tech_bias(spikein_input = spikein_input, alleleA = alleleA,
+                  alleleB = alleleB, pdf = TRUE)
```

2.3 Gene classification

SCALE adopts a Bayes framework that categorizes each gene into being silent, monoallelically expressed, and biallelically expressed (including biallelically bursty). Proportions of cells expressing A and B alleles and gene categories are returned. Results from the first 10 genes are shown below. For genome-wide results, parallel computing on HPC is recommenede.

```
> gene.class.obj = gene_classify(alleleA=alleleA[1:10,], alleleB=alleleB[1:10,])

Gene 1 : Hvcn1 , Biallelic.bursty         A prop 0.231 B prop 0.264
Gene 2 : Gbp7 , Silent         A prop 0 B prop 0
Gene 3 : Arrdc1 , Biallelic.bursty         A prop 0.23 B prop 0.197
Gene 4 : Ercc5 , Biallelic.bursty         A prop 0.358 B prop 0.183
Gene 5 : Mrpl15 , Biallelic.bursty         A prop 0.875 B prop 0.925
Gene 6 : Dclk1 , Silent         A prop 0 B prop 0
Gene 7 : Tssc4 , Biallelic.bursty         A prop 0.254 B prop 0.213
Gene 8 : Gm101 , Silent         A prop 0 B prop 0
Gene 9 : Pum2 , Biallelic.bursty         A prop 0.15 B prop 0.142
Gene 10 : Erv3 , Silent         A prop 0 B prop 0

> A.prop = gene.class.obj$A.prop # Proportion of cells expressing A allele
> B.prop = gene.class.obj$B.prop # Proportion of cells expressing B allele
> gene.category = gene.class.obj$gene.category # Gene category
> results.list = gene.class.obj$results.list # Posterior assignments of cells
```

2.4 Allele-specific bursting kinetics

The two alleles of a gene have two Poisson-Beta distributions with respective parameters. These two Poisson-Beta distributions share the same cell-size factor. Cell-size factor can be estimated by the expression level of *GAPDH* or by the ratio of total number of endogenous RNA reads over the total number of spike-in reads. A Poisson hierarchical model is used to account for technical variability that is introduced by sequencing and library prep. Histogram repiling method is used to adjust for technical variability (bandwidth is optimized based on correlations of the inferred kinetic parameters between the two alleles). Moment estimator is used to estimate bursting kinetics. A plot (pdf format) is generated by default as is shown in Figure 1.

```
> cellsize = rep(1, ncol(alleleA))  # cell size input
> allelic.kinetics.obj = allelic_kinetics(alleleA = alleleA[1:1000,],
+                                         alleleB = alleleB[1:1000,],
+                                         abkt = abkt,
+                                         gene.category = gene.category[1:1000],
+                                         cellsize = cellsize, pdf = TRUE)
```

```
Bandwidth  1 :       % non-neg estimates 0.859         corr. freq 0.897          corr. size 0.785
Bandwidth  2 :       % non-neg estimates 0.867         corr. freq 0.896          corr. size 0.793
Bandwidth  3 :       % non-neg estimates 0.87         corr. freq 0.897          corr. size 0.787
Bandwidth  4 :       % non-neg estimates 0.87         corr. freq 0.898          corr. size 0.793
Bandwidth  5 :       % non-neg estimates 0.88         corr. freq 0.896          corr. size 0.797
Bandwidth  6 :       % non-neg estimates 0.872         corr. freq 0.899          corr. size 0.782
Bandwidth  7 :       % non-neg estimates 0.88         corr. freq 0.892          corr. size 0.789
Bandwidth  8 :       % non-neg estimates 0.878         corr. freq 0.898          corr. size 0.792
Bandwidth  9 :       % non-neg estimates 0.878         corr. freq 0.899          corr. size 0.794
Bandwidth  10 :       % non-neg estimates 0.875         corr. freq 0.898          corr. size 0.789
```

```
> bandwidth = allelic.kinetics.obj$bandwidth
> konA = allelic.kinetics.obj$konA; konB = allelic.kinetics.obj$konB
> koffA = allelic.kinetics.obj$koffA; koffB = allelic.kinetics.obj$koffB
> sA = allelic.kinetics.obj$sA; sB = allelic.kinetics.obj$sB
> sizeA = sA/koffA; sizeB = sB/koffB
```

2.5 Hypothesis testing

Nonparametric hypothesis test and chi-square test are carried out to test whether the two alleles of a gene share the same bursting kinetics and whether they burst independently. For test of same burst size and burst frequency between the two alleles, there are two 'modes': the *raw* mode bootstrap-samples from the raw observed allelic read counts; the *corrected* mode bootstrap-samples from the adjusted allelic read counts. Both modes give very similar results while the latter runs faster.

```
> # Nonparametric test on whether the two alleles share the same burst frequency and burst size.
> diff.allelic.obj = diff_allelic_bursting(alleleA = alleleA,
+                                          alleleB = alleleB,
+                                          cellsize = cellsize,
+                                          gene.category = gene.category,
+                                          abkt = abkt,
+                                          allelic.kinetics.obj = allelic.kinetics.obj,
+                                          mode = 'corrected')
> pval.kon = diff.allelic.obj$pval.kon; pval.size = diff.allelic.obj$pval.size
```

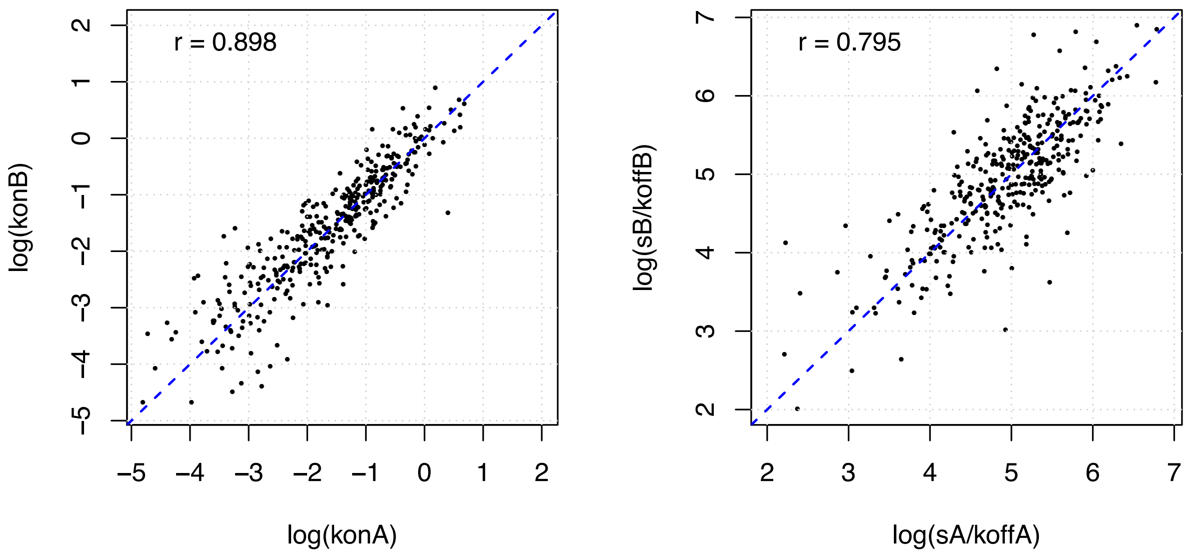

Figure 1: Allelic bursting kinetics (burst frequency and bursty size). Only first 1000 genes are computed.

```
> # Chi-square test on whether the two alleles fire independently.
> non.ind.obj = non_ind_bursting(alleleA = alleleA, alleleB = alleleB,
+                                gene.category = gene.category,
+                                results.list = results.list)
> pval.ind = non.ind.obj$pval.ind; non.ind.type = non.ind.obj$non.ind.type
```

2.6 Plot and output

For each gene, a plot (pdf format) can be generated with inferred parameters as well as summary statistics, as is shown in Figure 2.

```
> i=which(genename=='Btf3l4')
> allelic_plot(alleleA = alleleA, alleleB = alleleB,
+              gene.class.obj = gene.class.obj,
+              allelic.kinetics.obj = allelic.kinetics.obj,
+              diff.allelic.obj = diff.allelic.obj,
+              non.ind.obj = non.ind.obj, i= i)
```

The final output of `SCALE` is a tab delimited text file. The columns include: `genename` (gene name), `gene.category` (gene category), `konA` (burst frequency A), `konB` (burst frequency B), `pval.kon` (p-value of shared burst frequency), `sizeA` (burst size A), `sizeB` (burst size B), `pval.size` (p-value of shared burst size), `A_cell`, `B_cell`, `AB_cell`, `Off_cell` (number of cells with posterior assignment of A, B, AB, and Off), `A_prop` (proportion of cells expressing A allele), `B_prop` (proportion of cells expressing B allele), `p.ind` (p-value of burst independence), and `non.ind.type` (direction of non-independent bursting: 'C' is for coordinated bursting; 'R' for repulsed bursting).

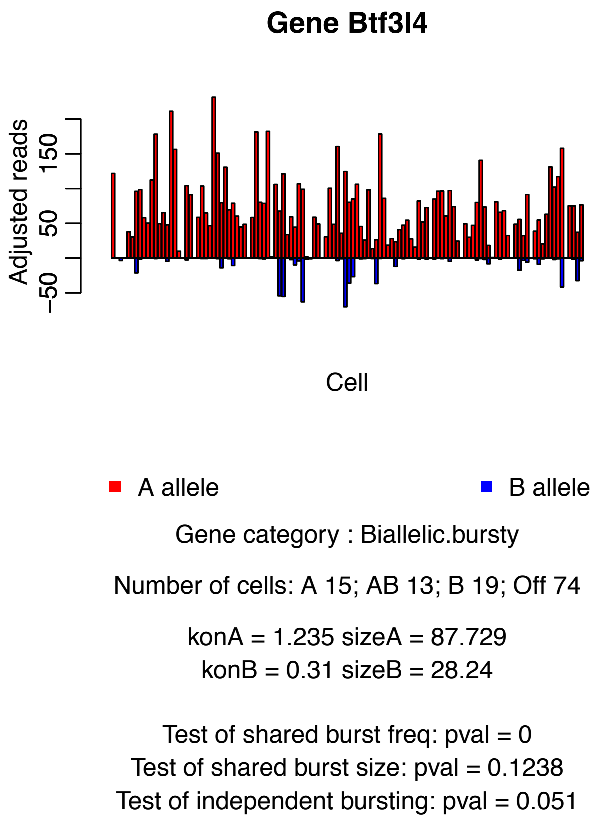

Figure 2: SCALE plot output for gene *Btf3l4*.

```
> SCALE.output=output_table(alleleA=alleleA, alleleB=alleleB,
+                           gene.class.obj = gene.class.obj,
+                           allelic.kinetics.obj = allelic.kinetics.obj,
+                           diff.allelic.obj = diff.allelic.obj,
+                           non.ind.obj = non.ind.obj)
> head(SCALE.output)

     genename gene.category      konA     konB     pval.kon  sizeA    sizeB
[1,] "Hvcn1"  "Biallelic.bursty" "0.08"   "0.0908" "0.6983"  "232.91" "263.37"
[2,] "Gbp7"   "Silent"           "-"      "-"      "-"       "-"      "-"
[3,] "Arrdc1" "Biallelic.bursty" "0.0825" "0.073"  "0.7166"  "199.31" "144.44"
[4,] "Ercc5"  "Biallelic.bursty" "0.0997" "0.0198" "0.05322" "322.53" "968.07"
[5,] "Mrpl15" "Biallelic.bursty" "1.2421" "1.3933" "0.72107" "150.31" "162.2"
[6,] "Dclk1"  "Silent"           "-"      "-"      "-"       "-"      "-"
     pval.size A_cell B_cell AB_cell Off_cell A.prop  B.prop  pval.ind
[1,] "0.7402"  "15"   "19"   "13"    "74"     "0.231" "0.264" "0.00624"
[2,] "-"       "0"    "0"    "0"     "122"    "0"     "0"     "-"
[3,] "0.50921" "18"   "14"   "10"    "80"     "0.23"  "0.197" "0.015"
[4,] "0.14033" "30"   "9"    "13"    "68"     "0.358" "0.183" "0.01181"
[5,] "0.86669" "5"    "11"   "100"   "4"      "0.875" "0.925" "0.00259"
[6,] "-"       "0"    "0"    "0"     "122"    "0"     "0"     "-"
     non.ind.type
[1,] "C"
[2,] "-"
[3,] "C"
[4,] "C"
[5,] "C"
[6,] "-"

> write.table(SCALE.output, file = 'SCALE.output.txt', col.names = TRUE,
+             row.names = FALSE, quote = FALSE, sep = '\t')
```

3. Citation

Modeling allele-specific gene expression by single-cell RNA sequencing, Yuchao Jiang, Nancy R zhang, Mingyao Li, *submitted*, 2016.

4. Session information:

Output of sessionInfo on the system on which this document was compiled:

- R version 3.2.3 (2015-12-10), `x86_64-apple-darwin13.4.0`
- Locale: `C/en_US.UTF-8/en_US.UTF-8/C/en_US.UTF-8/en_US.UTF-8`
- Base packages: base, datasets, grDevices, graphics, methods, stats, utils
- Other packages: SCALE 1.0.0, rje 1.9
- Loaded via a namespace (and not attached): tools 3.2.3
